# Effects of mechanical insufflation-exsufflation in preventing respiratory failure after extubation: a randomized controlled trial

**DOI:** 10.1186/cc11249

**Published:** 2012-03-15

**Authors:** Miguel R Gonçalves, Teresa Honrado, João Carlos Winck, José Artur Paiva

**Affiliations:** 1 Lung Function and Ventilation Unit, Pulmonology Department, University Hospital of São João, Faculty of Medicine, Av. Prof. Hernâni Monteiro, 4200-319 Porto, Portugal; 2 Intensive Care and Emergency Department, University Hospital of São João, Faculty of Medicine, Av. Prof. Hernâni Monteiro, 4200-319 Porto, Portugal

## Abstract

**Introduction:**

Weaning protocols that include noninvasive ventilation (NIV) decrease re-intubation rates and ICU length of stay. However, impaired airway clearance is associated with NIV failure. Mechanical insufflation-exsufflation (MI-E) has been proven to be very effective in patients receiving NIV. We aimed to assess the efficacy of MI-E as part of an extubation protocol.

**Method:**

Patients with mechanical ventilation (MV) for more than 48 hours with specific inclusion criteria, who successfully tolerated a spontaneous breathing trial (SBT), were randomly allocated before extubation, either for (A) a conventional extubation protocol (control group), or (B) the MI-E extubation protocol (study group). During the postextubation period (48 hours), group A patients received standard medical treatment (SMT), including NIV in case of specific indications, whereas group B received the same postextubation approach plus three daily sessions of mechanical in-exsufflation (MI-E). Reintubation rates, ICU length of stay, and NIV failure rates were analyzed.

**Results:**

Seventy-five patients (26 women) with a mean age of 61.8 ± 17.3 years were randomized to a control group (*n *= 40; mean SAPS II, 47.8 ± 17.7) and to a study group (*n *= 35; mean SAPS II, 45.0 ± 15.0). MV time before enrollment was 9.4 ± 4.8 and 10.5 ± 4.1 days for the control and the study group, respectively. In the 48 hours after extubation, 20 control patients (50%) and 14 study patients (40%) used NIV. Study group patients had a significant lower reintubation rate than did controls; six patients (17%) versus 19 patients (48%), *P *< 0.05; respectively, and a significantly lower time under MV; 17.8 ± 6.4 versus 11.7 ± 3.5 days; *P *< 0.05; respectively. Considering only the subgroup of patients that used NIV, the reintubation rates related to NIV failure were significantly lower in the study group when compared with controls; two patients (6%) versus 13 (33%); *P *< 0.05, respectively. Mean ICU length of stay after extubation was significantly lower in the study group when compared with controls (3.1 ± 2.5 versus 9.8 ± 6.7 days; *P *< 0.05). No differences were found in the total ICU length of stay.

**Conclusion:**

Inclusion of MI-E may reduce reintubation rates with consequent reduction in postextubation ICU length of stay. This technique seems to be efficient in improving the efficacy of NIV in this patient population.

## Introduction

The process of weaning from mechanical ventilation must balance the risk of complications due to unnecessary delays in extubation with the risk of complications due to early discontinuation and the need for reintubation [1]. Patients who require reintubation have been noted to have a significantly higher mortality rate than those who are successfully extubated on the first attempt [2].

Patients in the intensive care setting very often have impaired airway clearance. Endotracheal intubation prevents the patient from closing the glottis, which is necessary for effective coughing [3]. Care of the intubated patient includes direct suction applied through the endotracheal tube which clears a small portion of the airway, is ineffective for clearing secretions in the peripheral airways, and leaves the patient dependent on mucociliary clearance rather than on cough clearance [4]. Deep insufflation with a self-inflating ventilation bag can help, especially if accompanied by chest physiotherapy, but it does not recreate a cough [5].

Ventilator-associated pneumonia is an exceedingly common problem in intensive care units (ICUs), occurring in as many as 27% of patients [6]. The reasons for this are complex, but a significant role is played by the aspiration of upper-airway secretions and gastric contents [7]. Despite the many reports and reviews on ventilator-associated pneumonia, little emphasis is placed on airway clearance [8].

Predictors for successful weaning include respiratory rate less than 38 per minute and a rapid shallow breathing index below 100 breaths/min/L [9]. However, even in patients who satisfy weaning criteria and pass ventilator-weaning trials, an extubation failure rate of 10% to 20% exists [10].

Postextubation respiratory failure is defined as the presence of signs and symptoms of respiratory distress in the first 48 hours after extubation. The reasons given for extubation failure include lack of improvement in the work of breathing, hypoxemia, respiratory acidosis, retained secretions, and decreased consciousness [11]. However, if at extubation, the lungs are healthy and ventilation can be fully maintained noninvasively [12], then the only remaining concern is the effective expulsion of airway secretions [13]. Despite the importance of this factor, no ventilator weaning parameter addresses the ability to cough.

Early extubation, coupled with the use of noninvasive ventilatory support, has been used effectively to facilitate weaning [14,15], improve survival [16], decrease the incidence of ventilator-associated pneumonia [17,18], and reduce ICU length of stay [16,18]. However, a higher risk of NIV failure is seen when this is applied in patients that develop acute respiratory failure (ARF) after extubation, and the evidence that supports its application is controversial [19-21].

Despite a great interest in this field, the role of impaired airway-secretion clearance on the outcome of postextubation respiratory failure in critically ill ventilator-dependent patients is still not clear. Airway clearance may be impaired in disorders associated with abnormal cough mechanics, altered mucus rheology, altered mucociliary clearance, or structural airway defects. In recent years, new technologies and more advanced techniques have been developed to be more effective in secretion clearance for patients with acute respiratory failure [22-24].

Mechanical insufflation-exsufflation (MI-E) has been described as an efficient technique in patients with chronic muscle weakness [25-27]. It applies positive pressure followed by negative pressure across the entire airway (both central and peripheral), in contrast to direct tracheal suction, which applies negative pressure to a small, localized area [28]. This therapy is perhaps the most physiologic mechanical recreation of a natural cough, and we hypothesized that when applied after extubation, it may improve secretion clearance in critical ill respiratory patients and have positive outcomes on their postextubation condition.

The purpose of this study was to assess the efficacy of MI-E in preventing reintubation for patients in whom acute respiratory failure develops after extubation.

## Materials and methods

The data of this randomized control trial were gathered in a 12-bed general ICU between 2007 and 2009. The study was approved by the ethical committee of the São João University Hospital and institutional review board, and all the necessary participation consents were given.

### Patients

Patients older than 18 years and under mechanical ventilation for more than 48 hours, for acute hypoxemic and/or hypercapnic respiratory failure from a specific etiology, were evaluated for discontinuation of mechanical ventilation through the application of a spontaneous breathing trial (SBT).

The application of an SBT trial was considered appropriate when all of the following criteria were met: improvement of the condition that caused acute respiratory failure, respiratory rate less than 38 per minute, rapid shallow breathing index below 100 breaths/min/L [9], suspension of sedative medications, ability to stay alert and communicate, core temperature less than 38°C throughout the last 24 hours, suspension of vasoactive drugs, with the exception of dopamine at doses lower than 5 μg per kilogram of body weight per minute, and partial pressure of oxygen greater than 60 mm Hg on an inspired fraction of oxygen (FiO_2_) of 0.40 (PaO_2_/FiO_2 _>200) or less, with a positive end-expiratory pressure (PEEP) of 5 cm H_2_O.

Discontinuation of mechanical ventilation was assessed with a trial of spontaneous breathing with a T-tube on a FiO_2 _of 50% for up to 60 to 120 minutes. The SBT was considered successful when, during the trial time, none of the following symptoms were found: respiratory rate, > 35 beats/min; SpO_2 _< 90%; 20% variation of heart rate or blood pressure, respiratory distress, agitation, and loss of consciousness [29].

Patients who successfully tolerated one SBT were randomly allocated before extubation, by using a computer-generated table either for (A) conventional extubation protocol (control group) or (B) MI-E extubation protocol (study group). The randomization table and allocation sequence were concealed both from the primary investigator and from all medical, respiratory therapy, and nursing staff of the ICU. After each allocation, the primary investigator informed the staff of the group in which the patient was included.

During the first 48 hours after extubation, patients were observed for symptoms of acute respiratory distress/insufficiency as defined by dyspnea and the presence of at least two of the following: respiratory acidosis (pH < 7.35 with a PaCO_2 _>45 mm Hg), increased use of accessory respiratory muscles, increased respiratory rate (> 35 beats/min), and decreasing oxyhemoglobin saturation (that is, SpO_2 _< 90% and PaO_2 _< 80 mm Hg, with FiO_2 _> 50%.

Group A patients received (postextubation) standard medical treatment (SMT), including NIV in case of specific indications, whereas Group B received the same postextubation approach plus mechanical in-exsufflation (MI-E).

Patients with persistent weaning failure who failed three or more SBTs in 1 week were excluded. Exclusion criteria also included facial or cranial trauma, tracheostomy, active upper gastrointestinal bleeding, neurologic instability (inability to respond to direct simple orders), hemodynamic instability, lack of cooperation, and confirmed diagnosis of neuromuscular disease.

### Standard medical therapy

All patients received postextubation standard medical therapy, including supplemental oxygen, as needed, respiratory chest physiotherapy, bronchodilators, antibiotics, and any other therapies, as directed by the attending physician.

Criteria for NIV were the same in the two groups in the first 48 hours after extubation. Patients were submitted to NIV, if they met at least one of the following criteria, as judged after they had undergone the assigned treatment: respiratory rate, > 35 beats/min; SpO_2_, < 90%; 20% variation of heart rate or blood pressure; dyspnea with respiratory distress; PaO_2 _< 60 mm Hg; PaCO_2 _> 45 mm Hg; pH < 7.35. The failure of NIV was declared when the main criteria for its application were not solved or the patient showed intolerance to the technique in the first 2 hours of use.

Noninvasive ventilation was provided via an ICU ventilator with noninvasive mode or via a portable pressure-cycled ventilator through an oronasal mask as the first choice. Other interfaces, such as nasal, total face, helmet, and mouthpieces were used in case of patient's intolerance to the oronasal mask.

The fraction of inspired oxygen and the positive end-expiratory pressure were titrated to maintain the arterial oxygen saturation above 90% (or PaO_2 _> 60 mm Hg). The ventilator settings were subsequently adjusted as needed for the patient's comfort. The facial skin was assessed every 4 hours to prevent damage from the tightly fitting mask.

The decision regarding when to discontinue noninvasive ventilation was left to the attending physician.

### Mechanical insufflation-exsufflation protocol (study group)

After passing the SBT and being randomized to the study group, before extubation, all patients were submitted to a treatment of MI-E (three sessions) with the Cough Assist (Philips Respironics, Carlsbad, CA, USA) through the endotracheal tube with pressures set at 40 cm H_2_O for insufflation and -40 cm H_2_O for exsufflation pressure. An insufflation/exsufflation time ratio of 3:2 seconds and a pause of 3 seconds between each cycle were used. Eight cycles were applied in every session with an abdominal thrust timed to the exsufflation cycle.

In addition to the standard medical therapy, during the first 48 hours after extubation, each patient received three daily treatments by means of a lightweight, elastic oronasal mask. Treatments (three sessions each) were divided between morning, afternoon, and night, making a total of nine daily sessions (one before extubation and the others after extubation). No MI-E treatments were performed after the study period.

The daily treatment frequency and its outcomes were recorded in a diary by the nursing staff. All MI-E treatments were administered by a trained respiratory therapist, ICU physician, or nurse.

### Criteria for reintubation

In both patient groups, the final decision to reintubate was left to and made by the attending physician, who recorded the single most relevant reason for reintubation. Reason to reintubate was the existence of at least one of the following, as judged after the patient had undergone the assigned treatment: (a) respiratory or cardiac arrest; (b) respiratory pauses with loss of consciousness; (c) respiratory distress despite 2-hour treatment with SMT and NIV; (d) decreasing level of consciousness; (c) intolerance to NIV; (f) hypotension, with a systolic blood pressure below 90 mm Hg for more than 30 minutes despite adequate volume challenge, the use of vasopressors, or both; and (g) copious secretions that could not be adequately cleared or that were associated with severe hypoxemia. The final decision to reintubate was made by the attending physician, who recorded the single most relevant reason for reintubation. Only reintubations required in the first 48 hours after extubation were considered for this study.

### Outcomes

Reintubation rate was considered the primary end point. Total ICU length of stay, postextubation ICU length of stay, and NIV failure rate were considered secondary end points. Reasons for reintubation were also analyzed and compared between groups. A subgroup analysis, both for primary and secondary end points, was performed in patients that were submitted to postextubation NIV.

### Statistical analysis

Descriptive data are reported as mean ± standard deviation (SD). Statistical analysis was performed with SPSS software (Release 15.0; SPSS, Chicago, I1, USA).

#### Sample-size calculation

After reviewing the reintubation rates of similar respiratory patients within our ICU and the most recent literature on this topic, a baseline reintubation rate of 60% was estimated for this patient population. By using a type 1 error of 5%, we estimated that we would need 33 patients in each group to have a power of 80% to detect a reduction in reintubation rate to 30%. The latter was a rate that was suggested as reasonable from the available literature. In total, 75 patients were randomized. One interim analysis was performed after inclusion of 50% of the estimated patients by using an α curtailment (*P *< 0.005) to correct the analysis.

The primary end-point variable was to decrease reintubation rates, defined by the necessity of mechanical positive-pressure ventilation through orotracheal intubation in the first 48 hours after extubation, in the treatment group.

#### Comparisons between groups

For all end points, differences between groups were analyzed by using an unpaired Student *t *test for the parametric variables and an unpaired Mann-Whitney test for the nonparametric ones, respectively. *P *values were considered significant at < 0.05.

## Results

In total, 92 patients were considered for the study period, 17 of whom did not meet the inclusion criteria. Seventy-five patients (26 women) with a mean age of 61.8 ± 17.3 years old were randomized, 40 to the control group (group A) and 35 to the study group (group B). Baseline characteristics at baseline were similar in both groups, as well as the reasons for MV. Demographic data and patients' characteristics at the entry into the study are listed in Table 1.

**Table 1:** Baseline characteristics of patients at entry into the study

	Group A (*n *= 40)	Group B (MI-E) (*n *= 35)	*P*
Age (years)	62 ± 19.2	61.4 ± 15.1	NS
Sex (M/F)	21/19	28/7	NS
SAPS II	47.8 ± 17.7	45 ± 15	NS
Duration of MV (days)	9.4 ± 4.8	10.5 ± 4.1	NS
Patients with chronic pulmonary disorders (*n*, %)	9 (23%)	7 (20%)	NS
Patients with hypoxemic respiratory failure (n,%)	24 (60%)	18 (52%)	NS
			
Reasons for MV (*n*)			
COPD exacerbations	6	4	
Congestive heart failure	5	4	
Community-acquired pneumonia	11	6	
Hospital-acquired pneumonia	-	2	
Postoperative respiratory failure	5	8	
Acute lung injury	-	1	
Thoracic trauma	6	3	
Sepsis	7	4	
Cardiac arrest	-	3	

Data are presented as mean ± standard deviation. COPD, chronic obstructive pulmonary disease; MV, mechanical ventilation; NS, nonsignificant; SAPS II, New Simplified Acute Physiology Score.

During the 48 hours after extubation, none of the patients from both groups died, and 19 (48%) from group A and six (17%) from group B were reintubated. Twenty patients (50%) from group A and 14 patients (40%) from group B used NIV. All patients from both groups for whom NIV failed, according to the criteria, were reintubated. Considering this subgroup of patients, the reintubation rates related to NIV failure were significantly lower in the study group when compared with controls; two patients (6%) versus 13 (33%); *P *< 0.05, respectively.

Outcomes of both patient groups in the first 48 hours after extubation, including the reintubation rates, causes of reintubation, as well as ICU length of stay data, are listed in Table 2. When compared with controls, both duration of invasive mechanical ventilation and postextubation ICU length of stay were significantly shorter by 6 days (*P *< 0.05), and the reintubation rate was significantly lower (17% versus 48%) in the study group. No significant differences were noted in total ICU length of stay.

**Table 2:** Postextubation outcomes data

	Group A (*n *= 40)	Group B (MI-E) (*n *= 35)
NIV application, *n *(%) Reasons for NIV (*n*)	20 (50%)	14 (40%)
Respiratory rate > 35 beats/min	5 (25%)	9 (64%)
SpO_2 _< 90%	4 (20%)	1 (7%)
20% variation of HR or BP	1 (5%)	-
PaO_2 _< 60; PaCO_2 _>45	10 (50%)	4 (29%)
Total period of MV (days)	17.8 ± 6.4^a^	11.7 ± 3.5^a^
Patients reintubated (*n*, %)	19 (48%)^a^	6 (17%)^a^
		
Causes of reintubation (*n*)		
Respiratory pauses with loss of consciousness	-	1
Respiratory distress after 2-h NIV	6	2
Decreasing level of consciousness	2	-
Intolerance to NIV	2	-
Hypotension (systolic BP < 90 mm Hg for > 30 minutes)	-	1
Secretion encumbrance associated with severe hypoxemia	9	2
NIV failure rate, *n *(%)	13 (65%)^a^	2 (14%)^a^
Total ICU length of stay	19.3 ± 8.1	16.9 ± 11.1
Postextubation ICU length of stay	9.8 ± 6.7^a^	3.1 ± 2.5^a^

Data are presented as mean ± standard deviation. APS II, New Simplified Acute Physiology Score; COPD, chronic obstructive pulmonary disease; MV, mechanical ventilation; NIV, noninvasive ventilation; NS, nonsignificant. ^a^*P *< 0.05

## Discussion

The results found in this trial suggest that secretion management with MI-E may work as a useful complementary technique to prevent reintubation in patients in whom ARF develops in the first 48 hours after extubation. The use of MI-E had a stronger impact in preventing reintubation in the group of patients that required NIV, as shown by the lower NIV failure rates in the patients that were submitted to MI-E.

Significant debate still exists concerning the precise indications and efficacy of NIV in this patient population [30] without any mention of the problem of impaired airway secretion.

The randomized controlled studies performed by Esteban *et al. *[20] and Keenan *et al. *[19] concluded that NIV is not efficient in reducing reintubation rates, duration of invasive mechanical ventilation, and ICU and hospital length of stay. None of these studies showed improvement in survival in patients that used NIV in addition to SMT. On the contrary, the trials conducted by Nava *et al. *[31] and Ferrer *et al. *[32] showed that NIV could prevent ARF after extubation. Several facts may explain these differences.

First, whereas the studies by Keenan *et al. *[19] and Esteban *et al. *[20] applied NIV after patients had developed symptoms of respiratory failure, the studies by Nava *et al. *[31] and Ferrer *et al. *[32] previously identified the high-risk patients and applied NIV immediately after extubation. As a longer time from extubation to reintubation is associated with a worse outcome [33], delay in reintubation correlates with worse survival rates in patients who received NIV for established postextubation respiratory failure [19,20]. Thus, the early application of NIV seems crucial to avoid respiratory failure after extubation, and consequently reintubation.

Second, a significantly higher proportion of patients with chronic respiratory disorders were included in the studies that used early NIV in high-risk patients, whereas the NIV postextubation respiratory-failure trials enrolled only 10% to 11% of patients with chronic pulmonary disease and used different definitions for postextubation respiratory failure.

In our study, both study and control groups included 20% of patients with chronic respiratory disorders, and all patients were closely monitored during the first 48 hours for early detection of signs and symptoms that indicated postextubation respiratory failure. Similarly, in the studies performed by Esteban *et al. *[20] and Keenan *et al. *[19], NIV was applied only after the development of those symptoms, according to specific criteria. However, in our study, NIV was applied in both groups according to similar criteria, and only MI-E application was considered an independent variable for group B only.

Keenan *et al. *[19] showed a reintubation rate of 72% and 69% in the study (submitted to NIV) and the control group, respectively. Esteban *et al. *[20] also showed a high reintubation rate (48%) both in the study group and in the control group. The patients enrolled in these two studies were selected after confirmation of postextubation respiratory failure, which may justify such higher rates. Reintubation rates of the control group were also relatively high in our study, and although we enrolled a different population that underwent a planned extubation, this is a strong limitation of this study. However, our patient population included only patients with acute hypoxemic and/or hypercapnic respiratory failure with a considerable risk of extubation failure, and we used the same criteria for reintubation. Moreover, in our study, NIV was used in both groups, and its failure rate was also significant higher in group A (control group), a fact that also may justify the high reintubation rate in this group. The data in this study also confirm that extubation failure is associated with increased length of ICU stay.

In our study, the main reason for reintubation in group A was the presence of "secretion encumbrance associated with severe hypoxemia," in contrast with the fact that only two patients from group B were reintubated for the same reason. This fact may indicate that secretion clearance with MI-E might have had a positive role in preventing respiratory failure after extubation.

Secretion encumbrance with impaired airway clearance has been considered an independent factor for extubation failure [34], and associated with NIV failure both in persistent weaning [18] and in postextubation failure [20] patients. Although it is relatively easy to manage secretions through an endotracheal tube, it can be a serious problem after extubation and especially during NIV. Deep-airway-suction suctioning through the tube is a strategy most commonly used by nursing staff to manage secretions; while patients are receiving invasive MV, however, it can be traumatic, difficult to perform, and often ineffective in the extubated patients, because it must be performed blindly through the nose or the mouth.

This study randomly used MI-E (CoughAssist; Phillips Respironics International Inc., Murrysville, PA, USA) before extubation via invasive tube and after extubation through oronasal interface at sufficient inspiratory and expiratory pressures (minimum of 40 to -40 cm H_2_O) to expand fully and then to empty fully the adult lungs in 6 to 8 seconds, thereby expelling airway secretions while avoiding both hyper- and hypoventilation. The ability to use MI-E through the endotracheal tube immediately before extubation was the main intervention that permitted the study group patients to minimize the risk of postextubation secretion encumbrance, and, together with its three daily noninvasive applications, may have had an influence on the reduction of reintubation and NIV failure rates.

Although MI-E has been described as a very efficient technique in the acute setting for patients with neuromuscular disease (NMD), in the treatment of respiratory failure due to upper respiratory tract infections [28], to avoid intubation [35], and to facilitate extubation, decannulation, and to prevent postextubation failure [36-38], the evidence supporting the role of this technique in this other critically ill patient population is lacking. Indeed, this study is the first randomized controlled trial focused on MI-E in critical care.

The fact that strong evidence exists on the efficacy of MI-E in critically ill patients with NMD, and the authors' positive experience with this technique in this patient population, was the ethical reason to exclude them from the study. Our group recently reported a 97% extubation success with full-setting noninvasive ventilation (NIV) and MI-E in 157 NMD patients for whom previous extubations and/or SBTs had failed [39].

Potential limitations must be taken into account when analyzing the differences between the two groups. First, although no significant differences in baseline characteristics were found at the entry of the study between the two groups, hypoxemic respiratory failure was slightly more frequent in group A; this may had an impact on the NIV failure rate and consequent higher reintubation rate in this patient group, because NIV is more likely to fail and reintubation be required when severe hypoxia is present [40]. Second, six controls and four study group patients were reintubated immediately with no indication for NIV. This fact was associated with causes that could not be solved by MI-E, because cooperation with the technique is crucial in extubated patients. Third, ICU and hospital mortality was not analyzed, because the study design was limited to the analysis of the outcomes during the 48-hour postextubation period. This fact limits the interpretation of the data concerning long-term effects of MI-E in hospital mortality.

Another potential limitation of this type of open clinical trial is the difficulty for a correct blinding of the investigators that might lead to possible bias. Although we predefined the criteria for all relevant interventions, and clinical decisions to be made by the attending physicians, as well as the outcome variables, this bias could not be entirely controlled.

## Conclusions

Our results suggest that MI-E for secretion clearance is safe and efficient in ICU respiratory patients with indications for mechanical ventilation, and we recommend that this technique can be included proactively in an extubation protocol only for specific subgroups of patients that are candidates for a planned extubation, but may be at risk of being reintubated for respiratory failure after extubation. However, because of the paucity of data, and the fact that this is a pilot study performed in a center highly experienced with MI-E, more studies are required to settle the issue in other critically ill populations.

## Key messages

• Secretion encumbrance is one of the main reasons for respiratory failure after extubation.

• Mechanical insufflation-exsufflation (MI-E) is an efficient technique for cough augmentation in ICU respiratory patients, either through an endotracheal tube or through a facial mask.

• Mechanical insufflation-exsufflation (MI-E) can be efficient to manage copious secretions and help to avoid reintubation, as well as to improve NIV efficacy in patients that develop respiratory failure after extubation.

• Successful extubation protocols, defined as reintubation avoidance in the first 48 hours, have a positive impact in reducing postextubation ICU length of stay in respiratory patients.

## Abbreviations

FiO_2_: inspired fraction of oxygen; ICU: intensive care unit; MI-E: mechanical insufflation-exsufflation; MV: mechanical ventilation; NIV: noninvasive ventilation; NMD: neuromuscular disease; PEEP: positive end-expiratory pressure; SBT: spontaneous breathing trial; SMT: standard medical treatment.

## Authors' contributions

MRG wrote all drafts of the manuscript, gathered and analyzed the data, and actively participated in all the procedures of the study. TH was responsible for the elaboration and supervision of the extubation protocols, gathered data, and added material to the text of the manuscript. JCW contributed to the methodologic design of the study, statistical analysis, and revised and added material to the text of the manuscript. JAP oversaw and authorized all the procedures of the study, revised, and added material to the text of the manuscript. All authors read and approved the final manuscript.

## Competing interests

MRG and JCW received fees for lecturing and for attending professional meetings from Philips Respironics, Inc., which has an interest in the subject of this manuscript. TH and JAP declare that they have no competing interests.
